# Chemotherapeutic potential of betanin/capecitabine combination targeting colon cancer: experimental and bioinformatic studies exploring NFκB and cyclin D1 interplay

**DOI:** 10.3389/fphar.2024.1362739

**Published:** 2024-04-05

**Authors:** Rehab Ahmed, Sawsan A. Zaitone, Asmaa K. K. Abdelmaogood, Huda M. Atef, Mona F. M. Soliman, Alaa M. Badawy, Howaida S. Ali, AbdelNaser Zaid, Hatem I. Mokhtar, Lamiaa M. Elabbasy, Emad Kandil, Asmaa Mokhtar Yosef, Rama I. Mahran

**Affiliations:** ^1^ Department of Natural Products and Alternative Medicine, Faculty of Pharmacy, University of Tabuk, Tabuk, Saudi Arabia; ^2^ Department of Pharmaceutics, Faculty of Pharmacy, University of Khartoum, Khartoum, Sudan; ^3^ Department of Pharmacology & Toxicology, Faculty of Pharmacy, University of Tabuk, Tabuk, Saudi Arabia; ^4^ Department of Pharmacology & Toxicology, Faculty of Pharmacy, Suez Canal University, Ismailia, Egypt; ^5^ Department of Clinical Pathology, Faculty of Medicine, Suez Canal University, Ismailia, Egypt; ^6^ Department of Histology and Cell Biology, Faculty of Medicine, Mansoura University, Mansoura, Egypt; ^7^ Department of Medical Histology and Cell Biology, Faculty of Medicine, Mansoura University, Mansoura, Egypt; ^8^ Department of Medical Histology and Cell Biology, Faculty of Medicine, Horus University, New Damiettta, Egypt; ^9^ Department of Anatomy and Embryology, Faculty of Medicine, Mansoura University, Mansoura, Egypt; ^10^ Department of Pharmacology, Faculty of Medicine, University of Tabuk, Tabuk, Saudi Arabia; ^11^ Department of Pharmacology, Faculty of Medicine, Assiut University, Assiut, Egypt; ^12^ Department of Surgery, Faculty of Medicine, Jazan University, Jazan, Saudi Arabia; ^13^ Department of General Surgery, Faculty of Medicine, Assiut University, Assiut, Egypt; ^14^ Department of Pharmaceutical Chemistry, Faculty of Pharmacy, Sinai University-Kantara Branch, Ismailia, Egypt; ^15^ Department of Medical Biochemistry & Molecular Biology, Faculty of Medicine, Mansoura University, Mansoura, Egypt; ^16^ Department of Basic Medical Sciences, College of Medicine, Almaarefa University, Riyadh, Saudi Arabia; ^17^ Department of Surgery, Tulane University, School of Medicine, New Orleans, LA, United States; ^18^ PharmD Program, Faculty of Pharmacy, University of Tabuk, Tabuk, Saudi Arabia; ^19^ Department of Pharmacology, Faculty of Medicine, Suez Canal University, Ismailia, Egypt

**Keywords:** betanin, bioinformatic study, capecitabine, cyclin D1, experimental colon cancer, mouse, NFκB

## Abstract

**Introduction:** Betanin (C₂₄H₂₆N₂O₁₃) is safe to use as food additives approved by the FDA with anti-inflammatory and anticancer effects in many types of cancer cell lines. The current experiment was designed to test the chemotherapeutic effect of the combination of betanin with the standard chemotherapeutic agent, capecitabine, against chemically induced colon cancer in mice.

**Methods:** Bioinformatic approach was designed to get information about the possible mechanisms through which the drugs may control cancer development. Five groups of mice were assigned as, (i) saline, (ii) colon cancer, (iii) betanin, (iv) capecitabine and (v) betanin/capecitabine. Drugs were given orally for a period of six weeks. Colon tissues were separated and used for biological assays and histopathology.

**Results:** In addition, the mRNA expression of TNF-α (4.58-fold), NFκB (5.33-fold), IL-1β (4.99-fold), cyclin D1 (4.07-fold), and IL-6 (3.55-fold) and protein levels showed several folds increases versus the saline group. Tumor histopathology scores in the colon cancer group (including cryptic distortion and hyperplasia) and immunostaining for NFκB (2.94-fold) were high while periodic-acid Schiff staining demonstrated poor mucin content (33% of the saline group). These pathologic manifestations were reduced remarkably in betanin/capecitabine group.

**Conclusion:** Collectively, our findings demonstrated the usefulness of betanin/capecitabine combination in targeting colon cancer and highlighted that betanin is a promising adjuvant therapy to capecitabine in treating colon cancer patients.

## 1 Introduction

Colon cancer typically begins as a growth called a polyp on the inner lining of the colon that becomes cancerous overtime (Fabregas et al., 2022). Its characteristics include uncontrolled cellular growth and proliferation involving the colonic crypt epithelial lining, emerging in the form of hyperplasia and gradually developing into an invasive carcinoma (Almet et al., 2020). Many experimental animal models have been established for studying the molecular pathogenesis of colon cancer and testing preventive nutritional and pharmacologic agents (Alshaman et al., 2022). A 1,2-dimethylhydrazine (DMHZ) model is a common chemically induced animal model of dysplastic colon and aberrant cryptic foci (ACF) that are considered prerequisites for colon cancer, which is widely used by researchers for studying colon cancer pathogenesis (Attia et al., 2021).

Commonly used chemotherapies for treating colon cancer comprise capecitabine, 5-fluorouracil, trifluridine, tipiracil, and oxaliplatin (Gill et al., 2003). Capecitabine is a prodrug that is often used alone or in combinations to treat different types of malignancies (Feng et al., 2020), including colon cancer (Knikman et al., 2020). Capecitabine is a fluoropyrimidine antimetabolite agent. Its mechanism of action as an antimetabolite leads to cell cycle arrest and blockage of DNA polymerase. Its pharmacokinetic properties show inter-individual variability; this may be attributed to the variation in enzyme activity (Deng et al., 2015). Capecitabine has estrogenic, cytotoxic, and teratogenic properties (Huo et al., 2020). Toxic adverse effects of capecitabine also include hair loss, myelosuppression, and gastrointestinal problems (Fernández et al., 2021). Hence, toxicity may raise concerns about the utilized doses. It is well known that capecitabine is frequently combined with other drugs (Pouya et al., 2021; Kibudde and Begg, 2022) to treat cancer. Treating cancer with a combination of nutraceuticals and anticancer drugs is a good strategy (Singh et al., 2018; Milella et al., 2023). Nowadays, capecitabine in combination with other non-chemotherapeutic agents has also been proven effective (Ruiz-Rabelo et al., 2011; Lin et al., 2020).

Betalains are water-soluble, orally bioavailable pigments that are extracted from beetroots (Vieira Teixeira da Silva et al., 2019). Red beet pigments have been recognized for their multiorgan anticancer effects *in vitro* and *in vivo* (Kapadia and Rao, 2012) and reduced chemically induced esophageal tumor incidence (Lechner et al., 2010).

Betanin (C₂₄H₂₆N₂O₁₃, betanidin-5-O-β-glucoside) is the most common betacyanin pigment in the plant kingdom. Betanin is a safe component for use as a food additive and food coloring and is approved by the FDA and the European Union (Vieira Teixeira da Silva et al., 2019). Studies have shown potential health benefits of betanin, primarily as an anti-inflammatory agent (ElSayed et al., 2023a). Betanin has anticancer effects and inhibits inflammatory cytokines in microglial cells (Ahmadi et al., 2020). It shows significant anticancer effects in human hepatic cell lines (Krajka-Kuźniak et al., 2013), U87MG human glioma cells (Salimi et al., 2021), growth of the breast cancer cell line MCf7 (Reddy et al., 2005), and Caco-2 colon cancer cells (Zielińska-Przyjemska et al., 2016). In addition, betanin inhibits the proliferation of the human colon cancer cell line HCT116 (Reddy et al., 2005). Betanin significantly reduced tumor multiplicity and tumor load after oral administration in female A/J mice (Zhang et al., 2013) but was not tested previously *in vivo* in combination with capecitabine.

To date, the effect of combining betanin with capecitabine has not been pursued. In the present study, our aim was to test the chemopreventive activity of the betanin/capecitabine combination in a model of chemically induced colon cancer and explore the possible inhibition of NFκB signaling and cyclin D1 protein using bioinformatic tools.

## 2 Materials and methods

### 2.1 Rationale and bioinformatic evidence

The KEGG database-refined tool, KEGG MEDICUS (www.kegg.jp/kegg/medicus), was used to explore the genes and proteins interacting in colon cancer. This health-related information tool integrates the KEGG network, disease, and drug with drug categories (ElSayed et al., 2023b; Kanehisa et al., 2023). Colon cancer was used as the entry category in the search box. Chemical carcinogenesis—receptor activation pathway (Pathway ID: hsa05207)—best described and reflected the postulation of the current experiment.

Two other freely available databases were also used to incorporate more targets, namely, Online Mendelian Inheritance in Man (OMIM), available at https://omim.org/ (Amberger et al., 2015), and DisGeNET (v7.0), available at http://www.disgenet.org/, with the gda index set at ≥ 0.1 for the simplification and summarization of the results (Piñero et al., 2019). Gene/protein lists from previous entries were exported from all the datasets and combined with for the removal of duplicates. This was followed by an investigation via FunRich software version 3.1.3 (www.funrich.org) (Pathan et al., 2015; Mohammad et al., 2023a) to annotate and visualize the overlapping genes.

The PubChem database (https://pubchem.ncbi.nlm.nih.gov) was accessed on the 10th of November 2023 and was used to obtain the isomeric Simplified Molecular-Input Line-Entry System (SMILES) for betanin (phytolaccanin) (Kim et al., 2023). The SMILES served as the input in the search box for the SwissTargetPrediction tool of the Swiss Institute of Bioinformatics database (http://www.swisstargetprediction.ch/) (Daina et al., 2019), SuperPred database (http://prediction.charite.de) (Dunkel et al., 2008), and STITCH (v 5.0) database (http://stitch.embl.de) (Kuhn et al., 2008). These tools were used to create a list of proteins/genes that are most likely targeted by this novel drug, all of which were combined and the duplicates removed.

FunRich software version 3.1.3 (www.funrich.org) was used again to reveal the shared putative targets between those of colon cancer and betanin and compare, identify, and visualize the overlapping genes.

The overlapping genes from the two lists were pasted into the gene enrichment graphical tool ShinyGO 0.77 (bioinformatics.sdstate.edu/go) for the exploration of the enriched pathways and the incriminated targets that require investigations (Ge et al., 2020).

### 2.2 Animal environment

A total of 60 male Swiss albino mice (8–10 weeks old with a body weight of 21–29 g) were purchased from Mustafa Rashed Company (Cairo, Egypt). The mice were housed in plastic cages in a normal diurnal cycle and with food and water available *ad libitum*. Acclimatization to the housing conditions was performed for 1 week, and then, the mice were randomly assigned to five groups. The experimental procedures were performed following the National Research Council’s Guide for the Care and Use of Laboratory Animals, and all procedures were performed in compliance with relevant laws and institutional guidelines. The experimental procedures were approved by the Research Ethics Committee of the Faculty of Pharmacy (202211RA9) and the Faculty of Medicine (Research 4664#) at the Suez Canal University. All measures were undertaken to minimize animal suffering. The experimental reports were performed following the Animal Research: Reporting of *In Vivo* Experiments (ARRIVE) guidelines.

### 2.3 Experimental model

**FIGURE 1:**
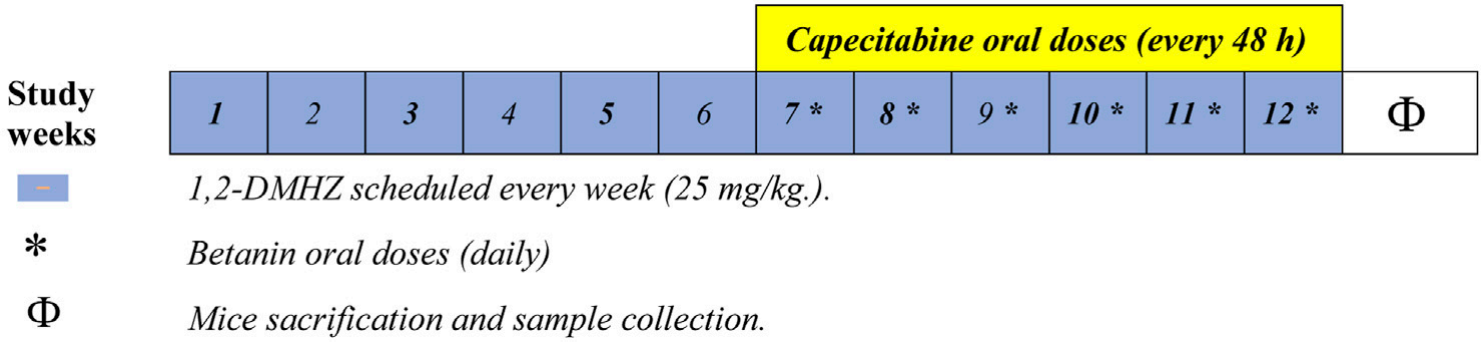
Diagram illustrating the experimental groups.

In this study, 1,2,-dimethylhydrazine (DMHZ; Sigma-Aldrich, United States) was used to induce colon cancer in mice, as reported previously (Hamiza et al., 2012; Bahr et al., 2021). In brief, DMHZ was dissolved according to the manufacturer’s instructions and diluted in phosphate-buffered saline (PBS) before use. The mice were randomly assigned to negative control and test groups (*n* = 12). The mice in the saline group received intraperitoneal injections of PBS once weekly for 12 weeks. The mice in the test group received the DMHZ solution (I.P.) once weekly for 12 weeks at 25 mg/kg. The other groups were divided into “colon cancer” control (DMHZ-treated for 12 weeks) and drug-treated groups (Figure 1). Betanin (901266-1G, molecular weight = 550.5 g/mol) was obtained from Sigma-Aldrich and dissolved in distilled water, while capecitabine was a product of Roche (California, United States).

The different groups can be summarized as follows:

Group I: Mice treated with PBS injections weekly at the same time as DMHZ injections.

Group II: Mice treated with DMHZ (I.P.) once weekly for 12 weeks (Hamiza et al., 2012; Bahr et al., 2021).

Group III: Mice treated with DMHZ (I.P.) weekly for 12 weeks and treated with oral doses of capecitabine (60 mg/kg; Roche) every 48 h for 6 weeks (Manu et al., 2014).

Group IV: Mice treated with DMHZ (I.P.) weekly for 12 weeks and treated with oral doses of betanin (50 mg/kg; Sigma-Aldrich, MO, United States) (ElSayed et al., 2023a) every day for 6 weeks.

Group V: Mice treated with DMHZ (I.P.) weekly for 12 weeks and treated with capecitabine and betanin—in the same aforementioned schedule—for 6 weeks.

In general, therapeutic regimens were administered by oral gavage starting from week 7 until the end of week 12. The animals were inspected daily to investigate any signs of distress. At the end of the experiment, the mice were anesthetized using ketamine and euthanized by cervical dislocation.

We rapidly dissected the descending colon, washed it with PBS, and divided it into two portions, as described previously (Attia et al., 2021). One portion was fixed in 10% paraformaldehyde for immunohistochemical analysis. Three other portions were quickly frozen at −80°C for further analysis.

### 2.4 ELISA assays for tumoral inflammatory proteins

Tumoral homogenates were obtained by homogenizing a frozen colon specimen in RIPA buffer and used to estimate TNF-α (SEA133Mu) and NFκB (SEB824Mu) levels using kits provided by Cloud-Clone Corp. (Katy, TX, United States). IL-1β (Cat. No. 432601, BioLegend, San Diego, CA, United States) and IL6 (201-02-0050, SunRedBio, China) kits were utilized to determine these parameters.

### 2.5 Quantitative real-time PCR analysis

#### 2.5.1 Total RNA extraction and assessment of its quality

From the colon tissue (50 mg) homogenate, the total RNA was extracted utilizing TRIzol (Invitrogen, United States). The quantity (RNA yield) and purity (A260/230 and A260/280 ratios) of the total RNA were measured using the NanoDrop ND-1000 spectrophotometer (Thermo Scientific, United States).

#### 2.5.2 Quantitative real-time PCR

Complementary DNA (cDNA) was synthetized from 1 μg RNA utilizing an Applied Biosystems High-Capacity cDNA Reverse Transcription Kit (United States). Real-time PCR was conducted using the SYBR Green Master Kit (Fermentas, United States) and Applied Biosystems software version 3.1 (StepOneTM, United States). Twenty microliters were utilized for the quantitative real-time PCR (qRT-PCR). A housekeeping control gene, glyceraldehyde 3-phosphate dehydrogenase (GAPDH), was utilized in order to normalize the expression of the genes and selected based on a previous study (Attia et al., 2021). The comparative Ct method (2^−ΔΔCT^) was employed for the calculation of the fold changes in gene expression (Livak and Schmittgen, 2001). These Ct values were calculated using StepOne Real-Time PCR detection software. Primer3 software (version 4.1.0) was used to allocate the primer sets, and the specificity of these sets was determined using the Primer-BLAST program (https://www.ncbi.nlm.nih.gov/tools/primer-blast/). Table 1 shows the list of primer sets.

**TABLE 1:** Primer sequence for genes tested in the colon samples.

Gene	Primer sequence	Product size	RefSeq
*NFκB*	Forward primer: GCT​CAG​CTT​GTG​AGG​GAT​CT; reverse primer: CCC​AAC​CCT​CAG​CAA​ATC​CT	150 bp	NM_001410442.1
*TNF-α*	Forward primer: ACG​GCA​TGG​ATC​TCA​AAG​AC; reverse primer: GTG​GGT​GAG​GAG​CAC​GTA​G	116 bp	NM_001278601.1
*IL-1β*	Forward primer: GCC​CAT​CCT​CTG​TGA​CTC​AT; reverse primer: AGG​CCA​CAG​GTA​TTT​TGT​CG	230 bp	NM_008361.4
*IL-6*	Forward primer: TAC​CAC​TTC​ACA​AGT​CGG​AGG​C; reverse primer: CTG​CAA​GTG​CAT​CAT​CGT​TGT​TC	116 bp	NM_001314054.1
*Cyclin D1*	Forward primer: AGT​GCG​TGC​AGA​AGG​AGA​TT; reverse primer: CAC​AAC​TTC​TCG​GCA​GTC​AA	238 bp	NM_001379248.1
*GAPDH*	Forward primer: CTC​TGC​TCC​TCC​TGT​TCG​AC; reverse primer: GCG​CCC​AAT​ACG​ACC​AAA​TC	121 bp	NM_002046.7

### 2.6 Histopathology and immunohistochemistry

The formalin-fixed tissues were dehydrated in ascending ethanol, followed by clearing tissues in xylene and embedding them in liquid paraffin wax. Cut sections (5 µm) were prepared and stained with hematoxylin and eosin (H&E) staining, Feulgen staining, and periodic acid Schiff (PAS) stain following standard protocols. PAS staining is usually used to stain neutral mucins. Mucins are mucopolysaccharides, which are long chains of sugar molecules found throughout the body, essential for life, and significant in maintaining the structural integrity of bone, cartilage, skin, elastic tissue, and membranes. They are important in cell growth as they help regulate the flow of nutrients between capillaries and cells and are known as the “glue of life.”

Additionally, the cut sections were subjected to antigen retrieval in the Tris-EDTA solution (pH 9) and blocked in 5% normal goat serum, as detailed previously (Mohammad et al., 2023b). Next, an anti-NFκB antibody (diluted as 1:100; Cat. #RB-1638-R7, Thermo Scientific, Fremont, CA 94538, United States) was added to colon sections, followed by the appropriate biotin-conjugated secondary antibodies (Catalog #BSB 0205, Mouse–Rabbit PolyDetector, DAB HRP Brown Detection System). Lastly, the tissue sections were stained with the DAB reagent and counterstained using Mayer’s hematoxylin (Ali et al., 2019). The slides were examined under a light microscope [Leica, Model: DM 1000, Wetzlar, Germany] equipped with a PC-driven digital camera [Leica, Wetzlar, Germany]. H&E-stained sections were blindly scored according to the dysplastic and histopathological changes, as detailed previously (Alshaman et al., 2022), whereas the Feulgen-stained area, PAS-stained goblet cell area, and immunohistochemically stained sections were examined and identified using ImageJ software (NIH, Bethesda, United States) following previously described procedures (Ali et al., 2015).

The general architecture of colon specimens stained with H&E was inspected, and imaging was done for A) colon dysplasia, B) hyperplasia, C) cryptic distortion, D) stromal cell infiltration (the degree of cell invasion into the connective tissue surrounding the tumor), and E) goblet cell depletion. ACF are defined as “clusters of abnormal tube-like glands in the lining of the colon and rectum.” ACF form before colorectal polyps and are one of the earliest changes that can be observed in the colon that may lead to cancer.

Blind scoring was performed according to 0–3 grading based on the severity of the findings. Next, the grand score was calculated for each group and presented as medians. In addition, we measured the area % of inflammatory filtrates and the thickness of mucosal layers in two sections per mouse and used six mice from each group. The images were analyzed on an Intel^®^ Core I7^®^-based computer using VideoTest Morphology^®^ software (Russia).

### 2.7 Statistical analysis

After data collection, they were presented as the mean ± SD. We set a *p*-value < 0.05 as the acknowledged significance level. The variances between the experimental groups were estimated using the ANOVA test, followed by Bonferroni’s test to show the pair-wise group comparisons.

## 3 Results

### 3.1 Bioinformatic study indicated a relation between chemical carcinogens

**FIGURE 2:**
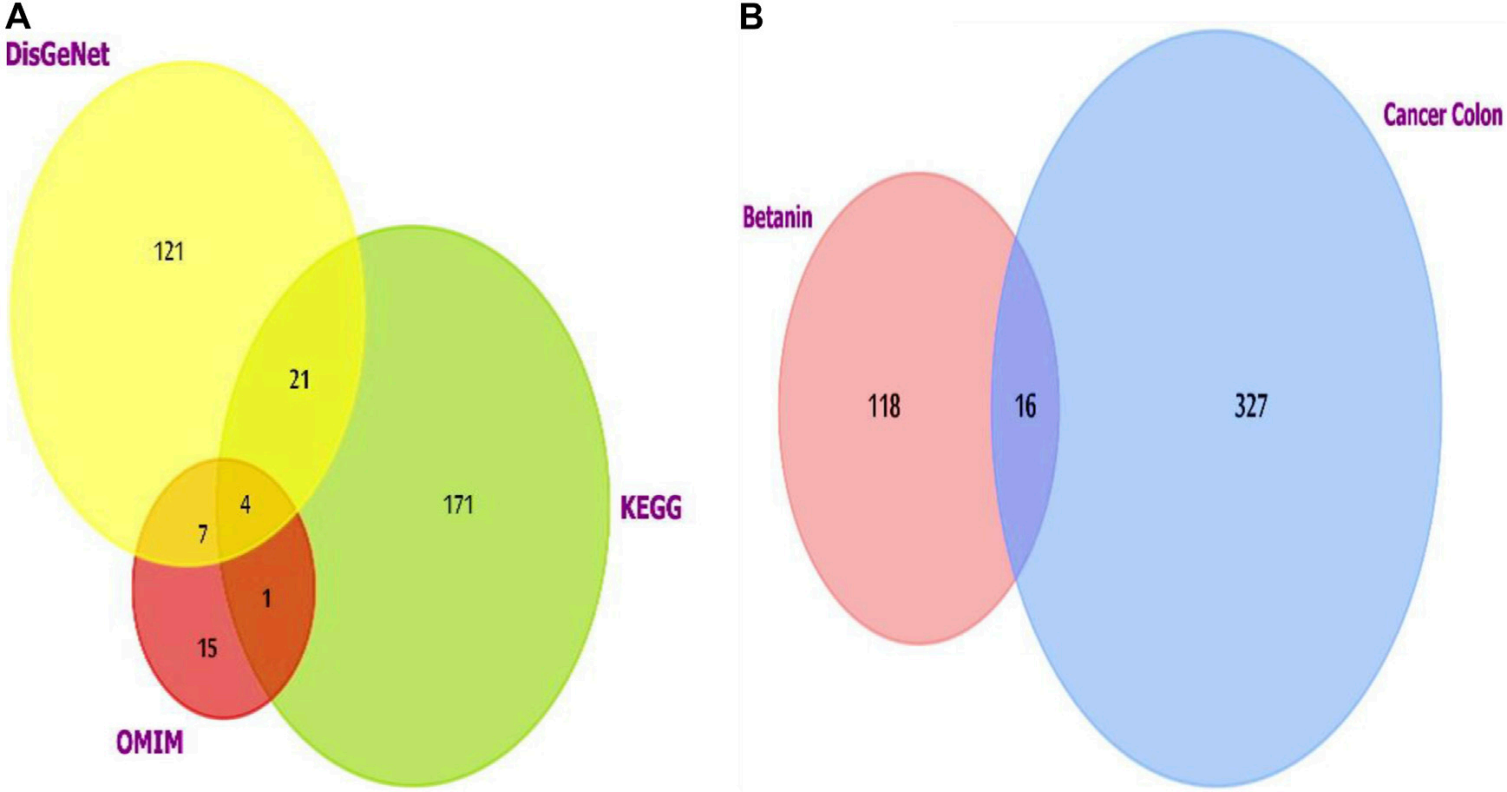
**(A)** Venn diagram showing the overlapping targets of the three databases used to investigate colon cancer-targeted proteins/genes. CCND1 was one of the four overlapped targets. **(B)** Venn diagram showing the shared targets of both colon cancer and betanin. Analysis and figure construction were done using FunRich software version 3.1.3.

For colon cancer, the list generated from the three datasets was investigated using FunRich software version 3.1.3, which revealed shared putative targets, and we compared the lists (Figure 2A). Four genes were consistent in the three databases (AKT1, CCND1, PIK3CA, and SRC).

G1/S-specific cyclin D1 (CCND1) was chosen for the downstream experiment due to its pivotal role in the cell cycle and carcinogenesis as it is a part of various cellular complexes that represent major integrators of many mitogenic and antimitogenic signals.

Colon cancer and betanin targets also showed intercepting shared targets, as explored using FunRich (Figure 2B). The 16 targets included many genes from which NFκB1 was chosen for estimation. It is a pleiotropic transcription factor existing in nearly all types of cells. Moreover, it is a cross-point in various signal transduction events that are triggered by chronic inflammation, apoptosis, and oncogenesis. TNF-α, IL-1β, and IL-6 are pivotal inducers of the NFκB complex and also one of its target genes; therefore, they will be included in future measurements. The addition of cyclin D will help understand the effect of the novel drug on cell cycle progression in normal and transformed scenarios through its relation to the growth-promoting effects of NFκB.

**FIGURE 3:**
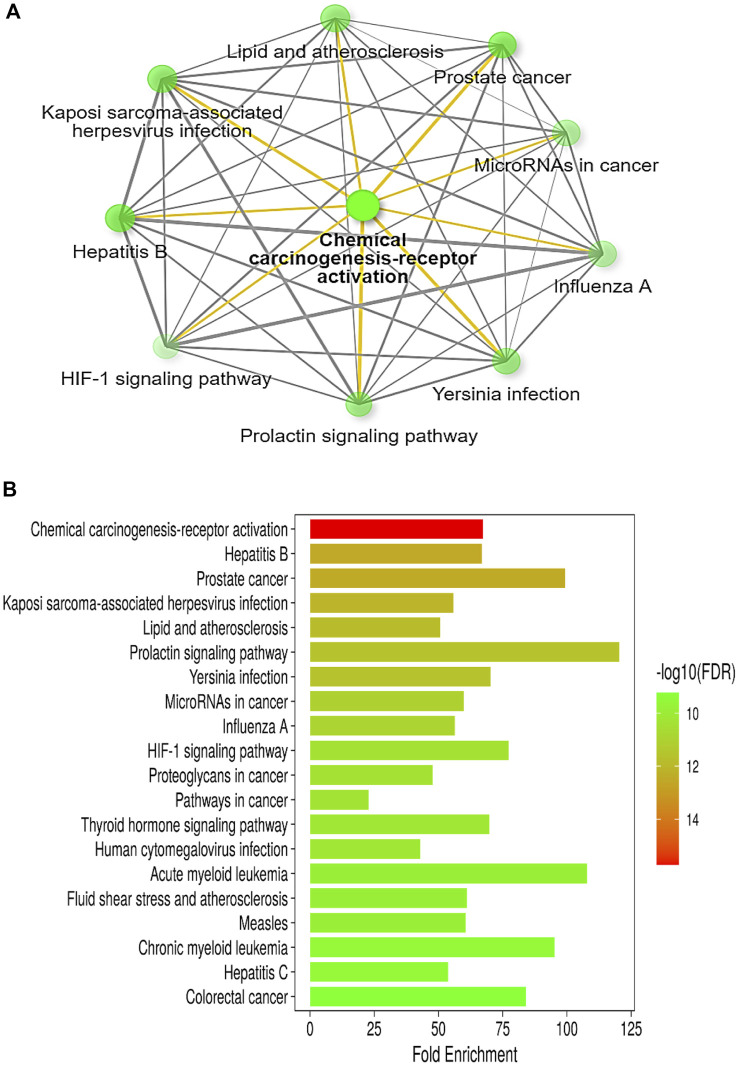
**(A)** Illustrative network of the top pathways (sorted by fold enrichment) targeted by colon cancer and betanin according to the KEGG pathway database. **(B)** A bar-plot chart of the top 20 pathways, sorted by FDR, enriched in both colon cancer and betanin-predicted target pathways. Nearly all the intercalating pathways are related to carcinogenesis and chronic insults. Both figures were constructed using the network tool, ShinyGO 0.77 software (bioinformatics.sdstate.edu/go).

The in-depth gene characteristic analysis and exploration of the enrichment using ShinyGO 0.77 of those inter-shared genes revealed intercalating and overlapping networks that all seemed to be strongly involved in the pathway proposed by our protocol as chemical carcinogenesis receptor activation pathways and microRNAs in cancer (Figures 3A, B).

### 3.2 Effects of the betanin/capecitabine combination on the inflammatory parameters

**FIGURE 4:**
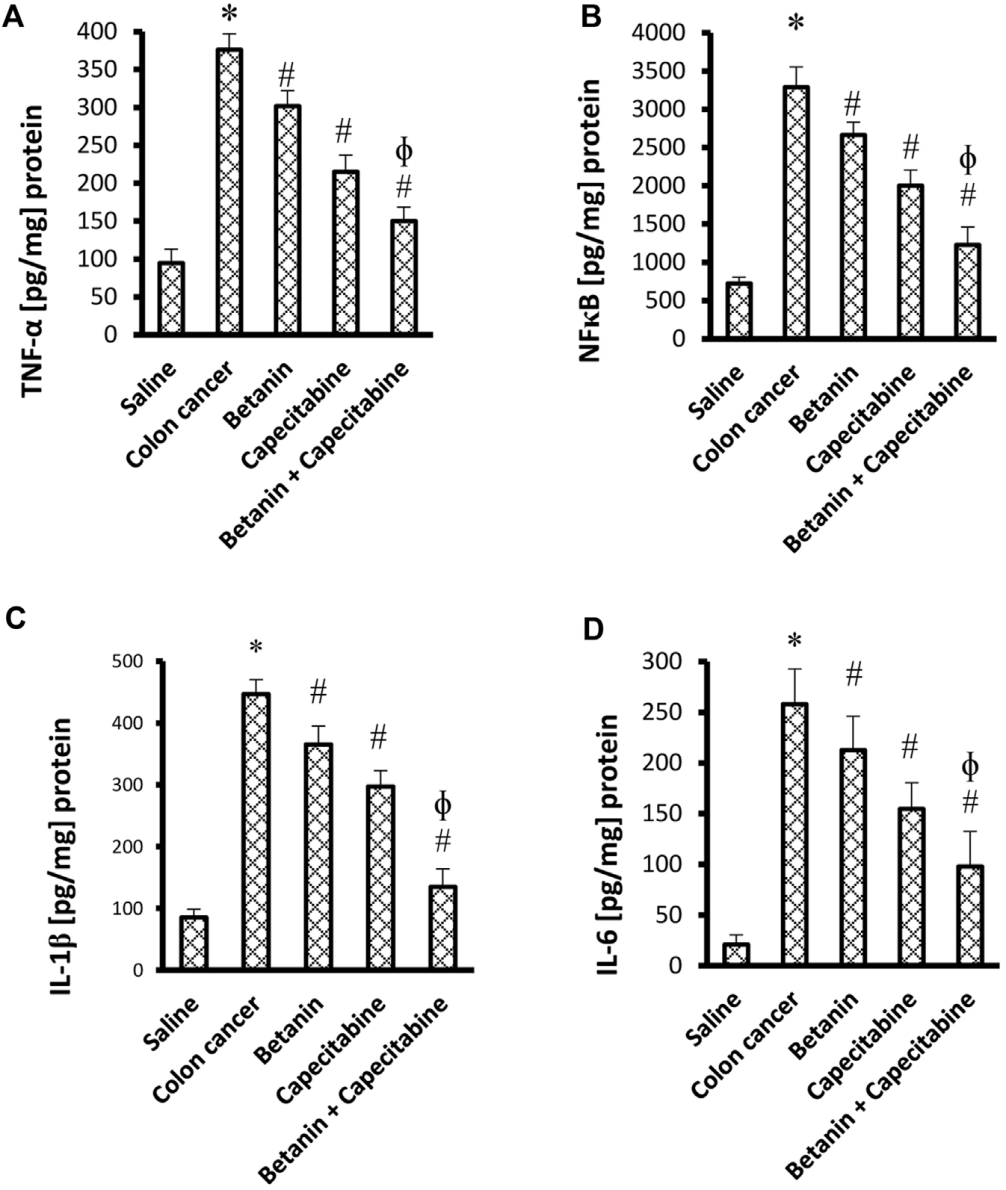
Impact of the betanin/capecitabine combination on tissue inflammatory parameters: **(A)** NFκB, **(B)** TNF-α, **(C)** IL-1β, and **(D)** IL-6. The data are presented as the mean ± SD and compared at *p* < 0.05. [*] versus saline control, [#] versus colon cancer control, and [Ф] versus the betanin group, *n* = 6.

The current results demonstrated elevated levels for TNF-α (376.2 ± 20.9 vs. 94.8 ± 18.3) and NFκB (3,287.7 ± 264.7 vs. 724.5 ± 79.1) (Figures 4A, B) and for the downstream products IL-1β (446.9 ± 23.3 vs. 85.25 ± 13.5) and IL-6 (258.0 ± 34.7 vs. 20.7 ± 9.8) (Figures 4C, D) in the colon cancer group versus the saline group. *Per se* treatment with betanin or capecitabine reduced the level of these inflammatory parameters compared to the colon cancer group. Interestingly, the betanin/capecitabine combination suppressed these inflammatory parameters to a greater extent than the betanin *per se* treatment (*p* < 0.05; Figures 4A–D).

### 3.3 mRNA expression of the inflammatory markers and cyclin D1

**FIGURE 5:**
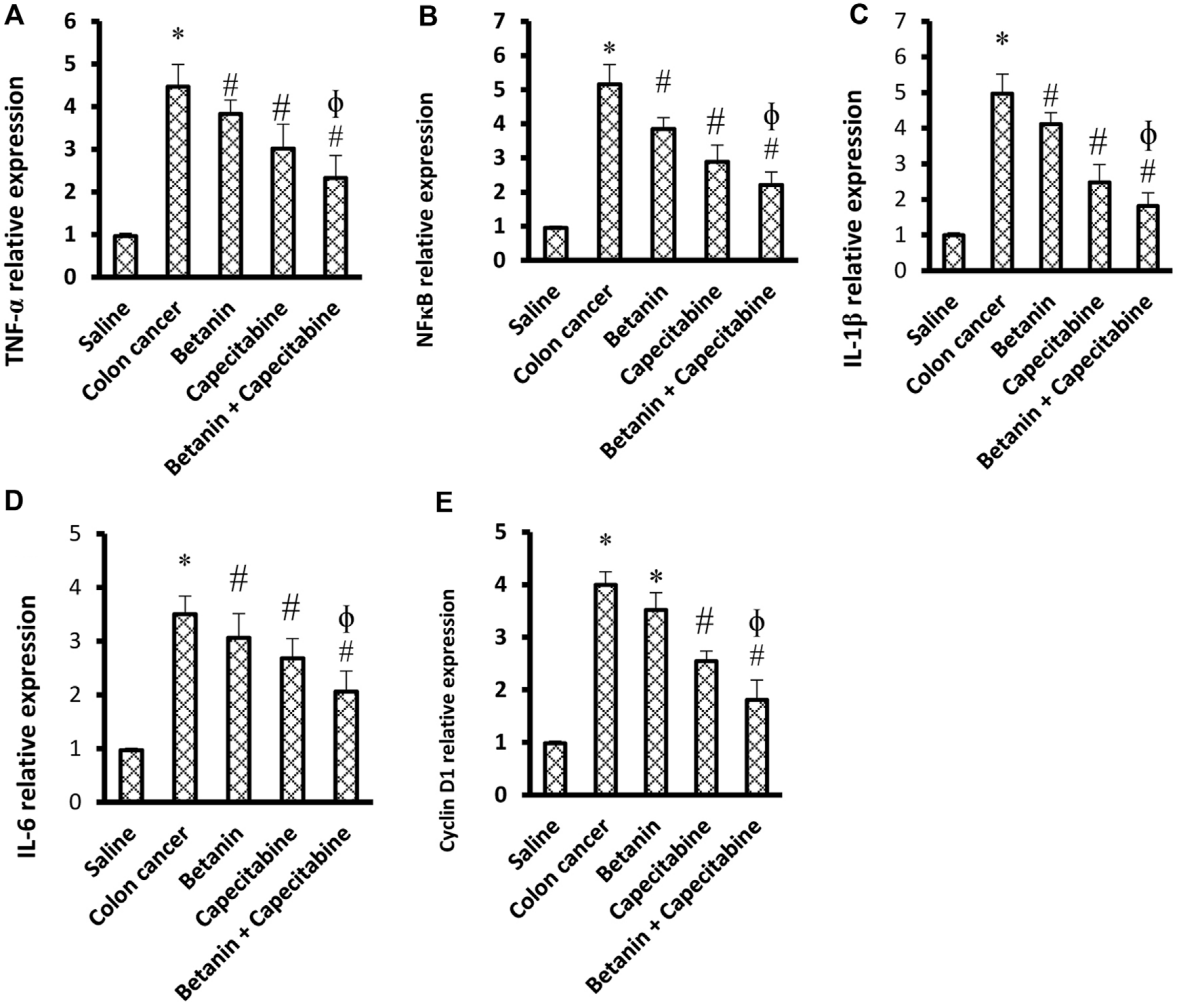
Impact of the betanin/capecitabine combination on tissue inflammation parameters and cyclin D1 in colon tissue specimens: **(A)** TNF-α, **(B)** NFκB, **(C)** IL-1β, **(D)** IL-6, and **(E)** cyclin D1. Data are presented as the mean ± SD and compared at *p* < 0.05. [*] versus saline control, [#] versus colon cancer control, and [Ф] versus the betanin group.

Figures 5A–E show significant increases in the mRNA expression of TNF-α (4.58-fold), NFκB (5.33-fold), IL-1β (4.99-fold), cyclin D1 (4.07-fold), and IL-6 (3.55-fold) in the colon cancer group compared to the saline group (*p* < 0.05; Figures 5D–G). Therapeutic doses of the betanin/capecitabine combination significantly downregulated the mRNA expression of the parameters TNF-α (Figures 5A), NFκB (Figures 5B), IL-1β (Figures 5C), IL-6 (Figures 5D), and cyclin D1 (Figures 5E) compared to the colon cancer control.

### 3.4 Colon histopathology and tumor score

**FIGURE 6:**
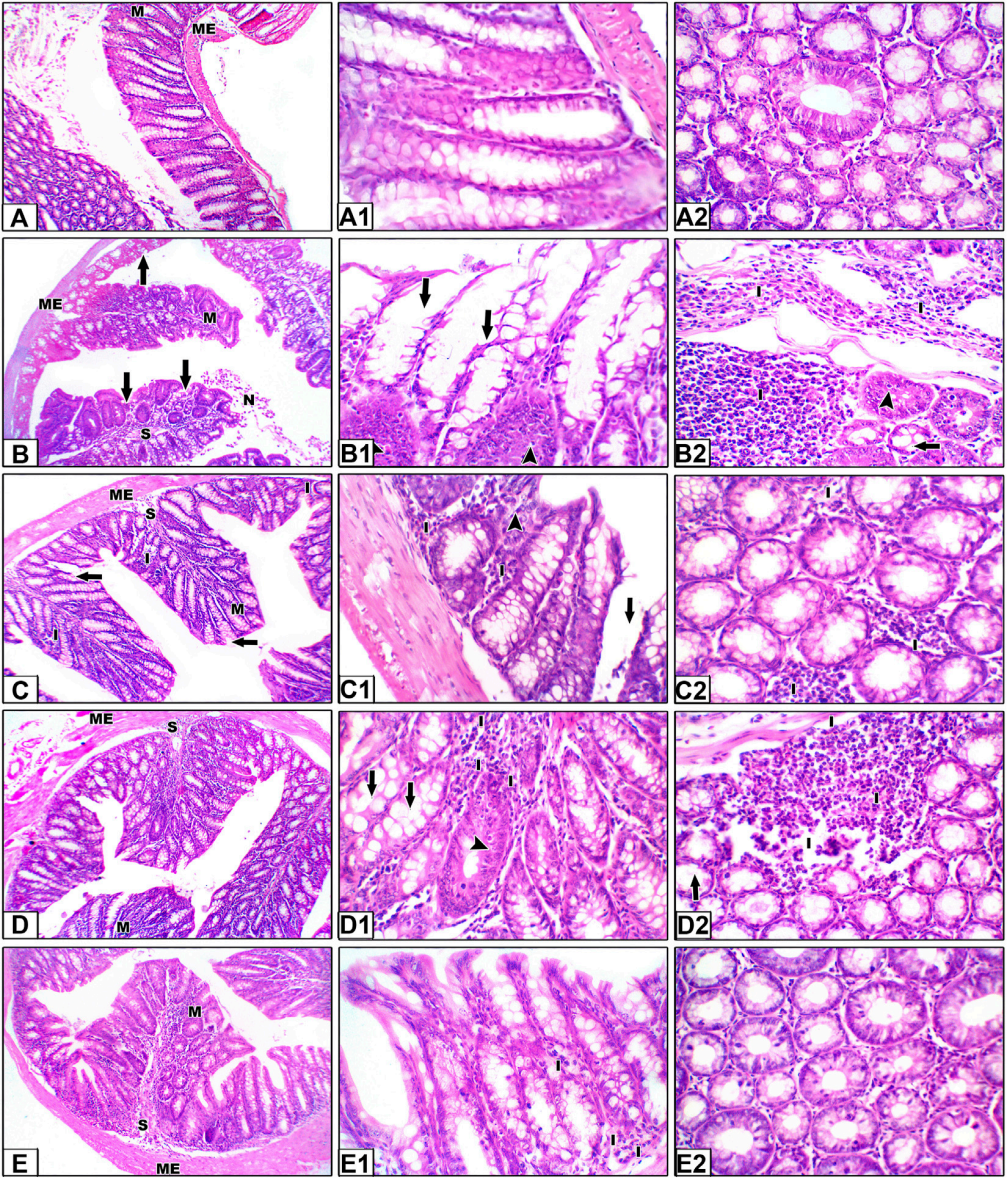
Hematoxylin and eosin staining for colon specimens. Panels **(A–A2)** Saline control group showing the mucosal layer (M) with normal-shaped crypts **(C)** lined by simple columnar epithelia with goblet cells, with minimal free lymphocytes in between. The submucosal connective tissue layer (S) is followed by the muscularis externa (ME). Panels **(B–B2)** Sections from the colon cancer group, distorted crypts lined by a dysplastic epithelium and goblet cells (arrows). Some crypts show hyperplastic changes (head arrows). Areas of dirty necrosis are observed in the lumen. A severe inflammatory infiltrate is noted in the connective tissue of the lamina propria and submucosa, even infiltrating the muscularis layer (I). Panels **(C–C2)** Sections from the capecitabine group show some disrupted crypts and a mild inflammatory infiltrate (I). Panels **(D–D2)** Sections from the betanin group showing few disrupted crypts and a moderate inflammatory infiltrate (I). Panels **(E–E2)** Sections from the betanin/capecitabine group showing normal crypts with minimal inflammatory infiltrate (I). Power × 100 **(A, B, C, D, E)** and power × 400 **(A1, A2, B1, B2, C1, C2, D1, D2, E1,** and **E2)**.

Hematoxylin and eosin staining shown in Figure 6 demonstrate colon specimens from the saline group. Panels A–A2 show H&E staining for sections from the saline control group. Colon sections of the saline group showed the mucosal layer containing many tubular intestinal glands cut transversely or longitudinally; the glands extend as deep as the muscularis mucosa. The submucosal layer consists of well-vascularized connective tissue, followed by the muscularis externa of the inner circular and outer longitudinal layers (ME). Panels B–B2 show sections from the colon cancer group showing hyperplasia and irregular-shaped mucosa lined by a dysplastic epithelium of hyperchromatic nuclei and columnar cells with pyknotic nuclei. Our results showed histological changes in the crypt glands, defined as hyperplastic and dysplastic changes. This is obviously shown in the H&E-stained sections (B1–B2) and in Supplementary Figure S1.

In panels C–C2, H&E staining for sections from the capecitabine group show moderate dysplasia of the epithelial lining, with some disrupted crypts and severe inflammatory infiltrates, whereas sections from the betanin group show partial dysplasia of the epithelial lining, with few disrupted crypts and moderate inflammatory infiltrates (Panels D–D2). Finally, H&E staining for sections from the betanin/capecitabine group show minimal dysplasia of the epithelial lining and normal crypts. Few inflammatory infiltrates are observed (Panels E–E2).

**FIGURE 7:**
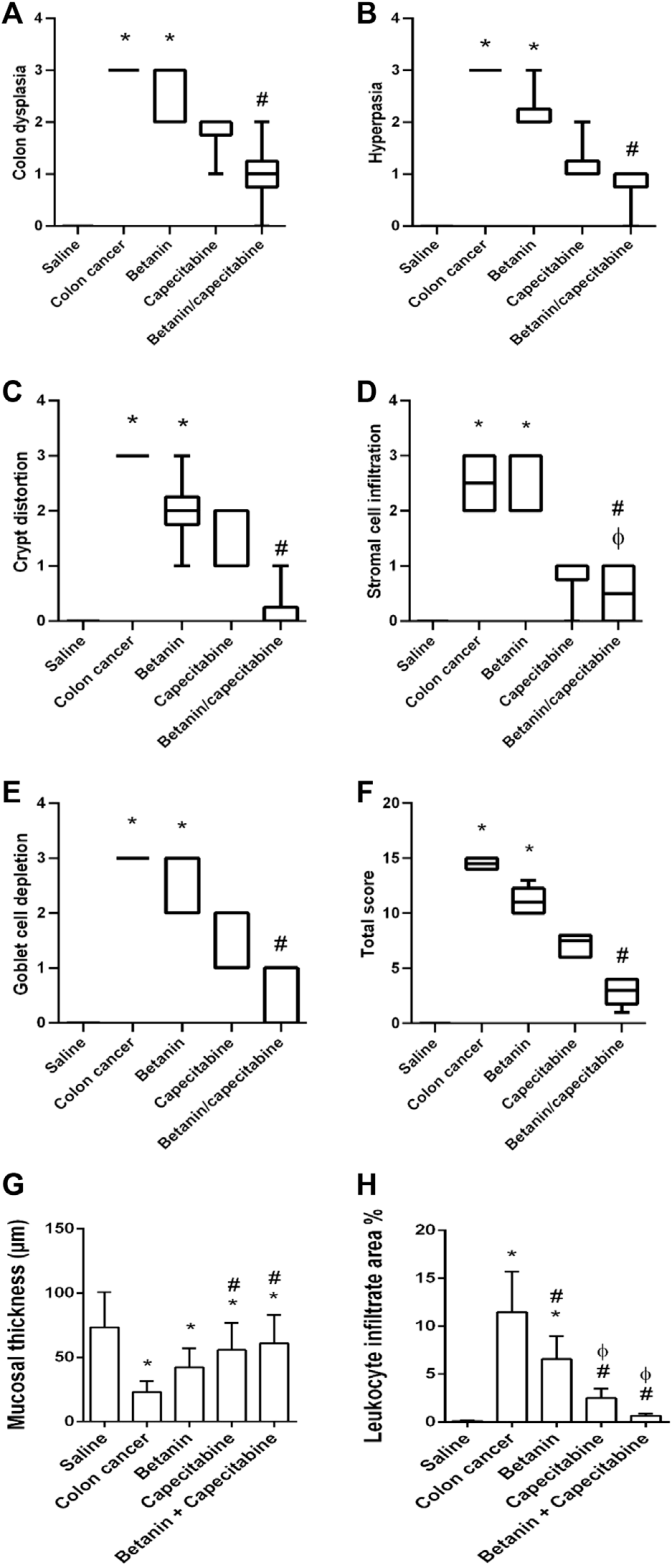
Scores of histopathology findings in colon sections in the experimental groups. **(A)** Colon dysplasia, **(B)** hyperplasia, **(C)** cryptic distortion, **(D)** stromal cell infiltration, **(E)** goblet cell depletion, **(F)** total histopathology, **(G)** mucosal thickness, and **(H)** leukocyte infiltrate area%. Data from six mice are graphically presented as boxplots, demonstrating the median value and the quartiles. [*] versus saline control, [#] versus colon cancer control, and [Ф] versus the betanin group at *p* < 0.05.

Furthermore, histopathologic scoring indicated greater scores for colon dysplasia (Figure 7A), hyperplasia (Figure 7B), cryptic distortion (Figure 7C), stromal cell infiltration (Figure 7D), goblet cell depletion (Figure 7E), and total histopathology (Figure 7F) in the colon cancer group than those in the saline group. Treatment with the betanin/capecitabine combination produced significant reductions in the individual scores and the total score compared to the colon cancer group (*p* < 0.05; Figure 7). In addition, the colon cancer group showed high mucosal thickness and leukocyte infiltrated compared to the saline group.

**FIGURE 8:**
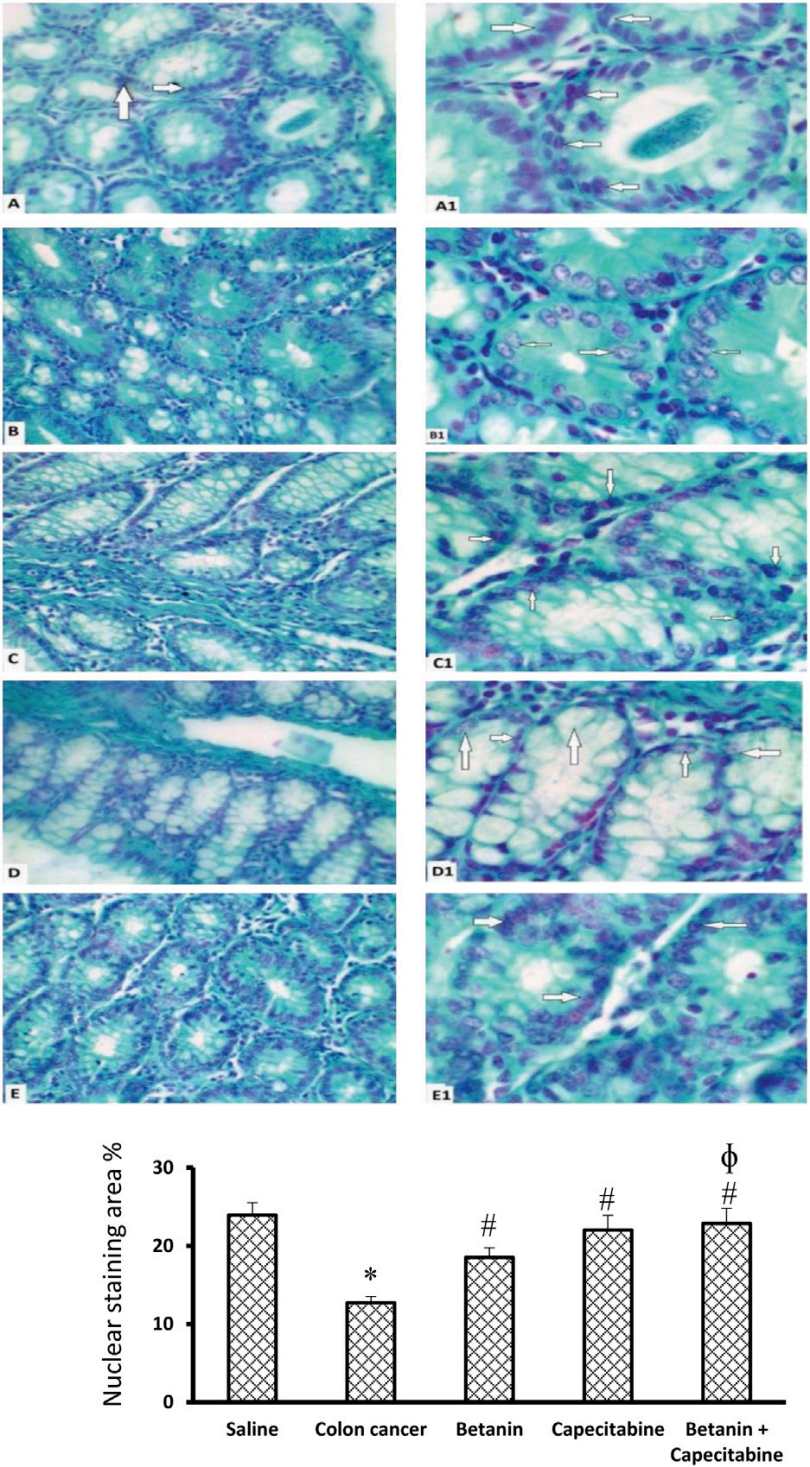
Microscopic images of colon sections stained with Feulgen stain. Photomicrograph of Feulgen-stained colon sections showing nuclei (white arrows) of cell lining crypts of the saline group **(A, A1)** dark-stained nuclei with regular shape and arrangement. The colon cancer control group **(B, B1)** showing faint-stained nuclei with some pyknotic nuclei, and others are fragmented. The capecitabine group **(C, C1)** shows some cells with dark-stained nuclei (white arrows) and others with faint-stained nuclei. The betanin group **(D, D1)** shows more faint-stained nuclei (white arrows) than the normal group, whereas the betanin/capecitabine group **(E, E1)** shows a marked increase in the number of dark-stained nuclei (white arrows). Power × 400 **(A, B, C, D, E)** and ×1,000 **(A1, B1, C1, D1, E1)**. **(F)** Column charts for the PAS-positive stained area. Data from six mice are graphically presented as boxplots, demonstrating the median value and the quartiles. [*] versus saline control and [#] versus colon cancer control at *p* < 0.05.

Figure 8 shows microscopic images of the colon sections stained with Feulgen stain, which represents nuclear staining for DNA. Feulgen-stained colon sections in the saline group show dark-stained nuclei of cell lining crypts (Figures 8A,A1) in a regular arrangement. The colon cancer control group (Figures 8B,B1) shows faint staining with nuclei showing pyknosis or fragmentation. The capecitabine group (Figures 8C, C1) shows some cells with dark nuclei and others with faint nuclei. The betanin group (Figures 8D, D1) shows more faint-stained nuclei than the saline group, whereas the betanin/capecitabine group (Figures 8E, E1) shows a marked increase in the number of dark-stained nuclei. Panel 8F represents the mean area of staining in the groups and shows that the betanin/capecitabine group produced a significant increase in Feulgen staining in nuclei compared (*p* < 0.05) to both the colon cancer group and betanin monotherapy group.

**FIGURE 9:**
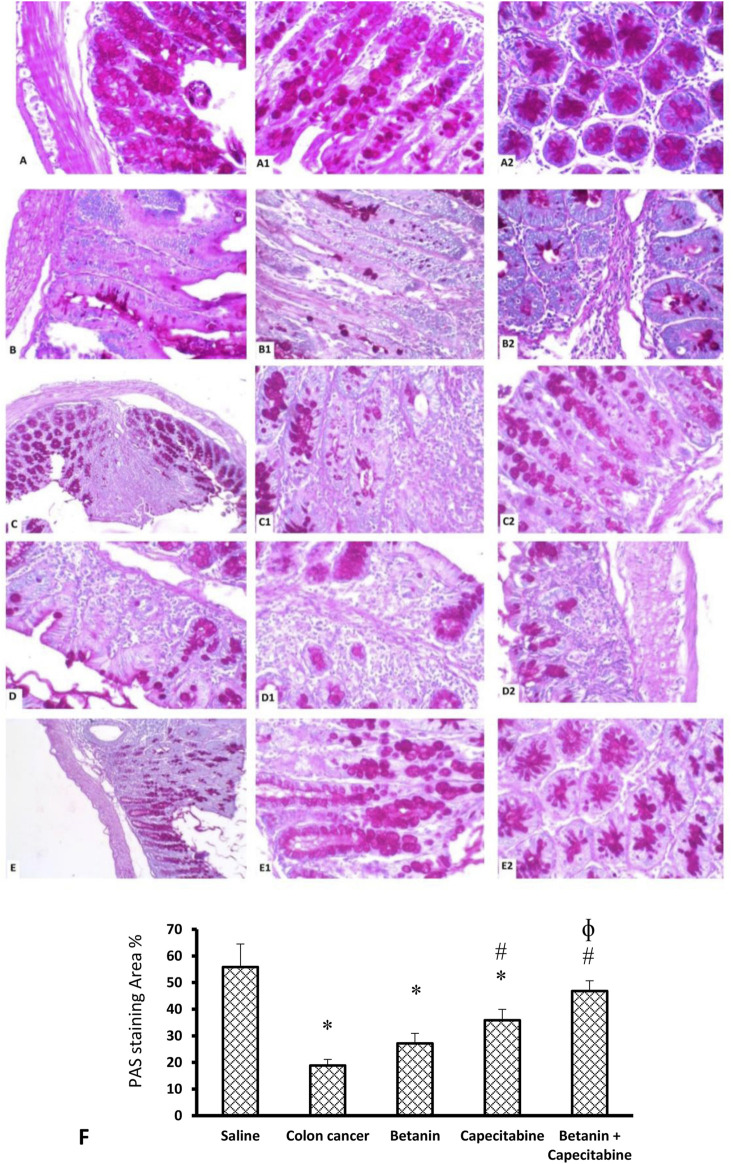
Microscopic images for colon sections stained with periodic acid Schiff stain. Panels **(A, A1, A2)** Saline group revealed abundant staining of PAS-positive goblet cells. Panels **(B, B1, B2)** Colon hyperplasia group shows a marked reduction in the number of stained PAS-positive goblet cells. Panels **(C, C1, C2)** The capecitabine group displays an increased number of positive goblet cells compared to the colon cancer control. Panels **(D, D1, D2)** The betanin-treated group shows a mild number of stained-positive cells. Panels **(E, E1, E2)** show a marked increase in positive-stained goblet cells in the betanin/capecitabine group. **(A–E)** Power × 100 **(A1, B1, C1, D1, E1)** and × 400 **(A2, B2, C2, D2, E2)**. **(F)** Column charts for the PAS-positive stained area. Data from six mice are graphically presented as boxplots, demonstrating the median value and the quartiles. [*] versus saline control and [#] versus colon cancer control at *p* < 0.05.

Figure 9 shows the PAS reaction in the mucosal goblet cells. The saline group revealed abundant PAS-positive staining in goblet cells (Panels 9A, 9A1, 9A2). The colon cancer group showed a marked reduction in the number of stained PAS-positive goblet cells (Panels 9B, 9B1, 9B2). The capecitabine group displayed a reduced number of positive goblet cells (Panels 9C, 9C1, 9C2). The betanin-treated group showed a moderate number of stained positive cells (Panels 9D, 9D1, 9D2), and the betanin/capecitabine group showed marked increases in positive-stained goblet cells (Panel 9E, 9E1, 9E2). The mean area of PAS staining was enhanced in the betanin/capecitabine group compared (*p* < 0.05) to both the colon cancer group and betanin monotherapy group (Panel 9F).

**FIGURE 10:**
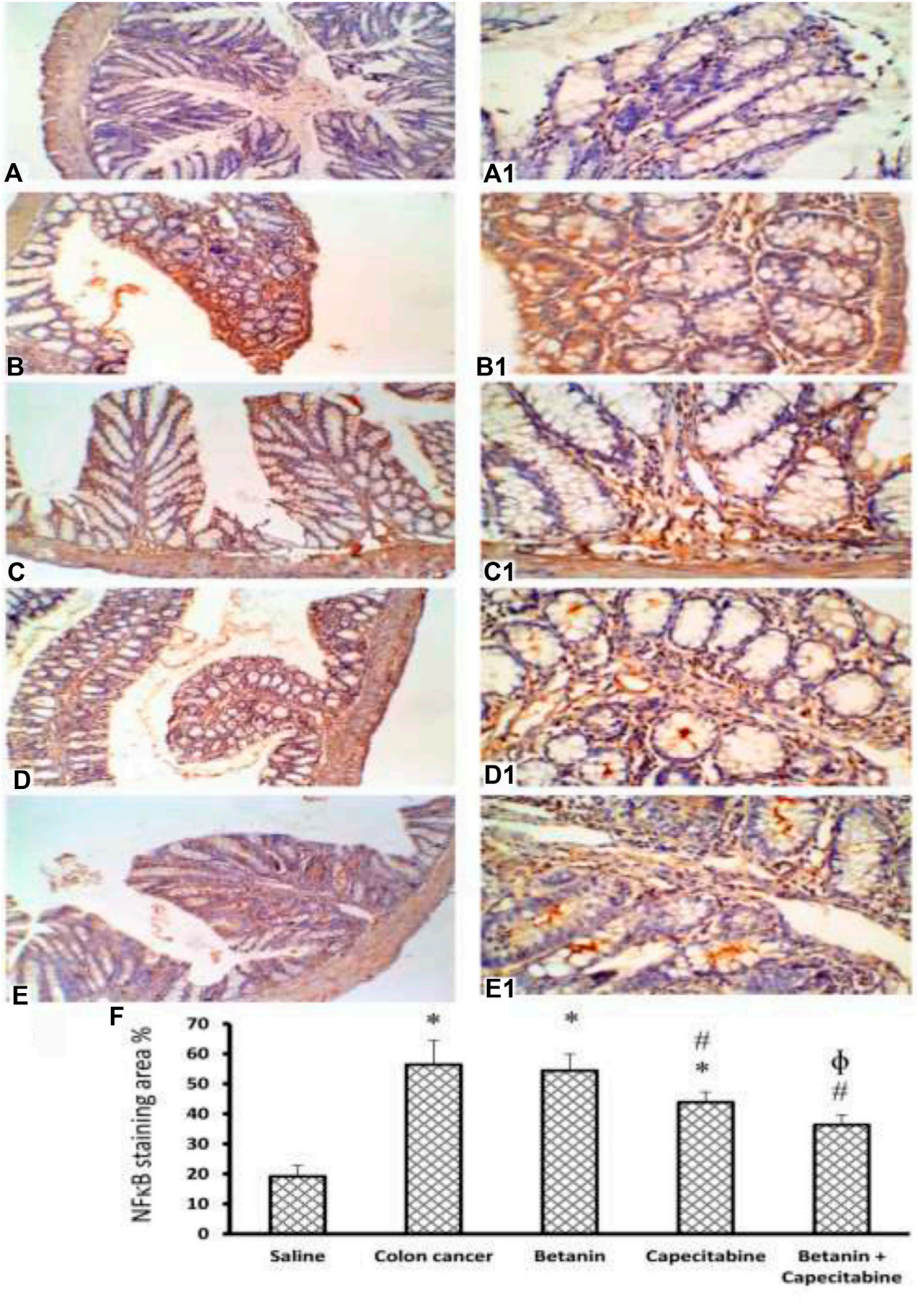
Microscopic images for colon sections stained for NFκB. Panels **(A, A1)** Photomicrograph from the saline group shows negative staining. Panels **(B, B1)** Photomicrograph of immunostained colon sections from the colon cancer control group shows a strong reaction within the hyperplastic colon. Panels **(C, C1)** Photomicrograph of the capecitabine group shows a mild positive reaction compared to other groups **(D, D1)**. Panels **(E, E1**) Photomicrograph of immunostained colon sections from the betanin/capecitabine group shows minimal reaction compared to other groups. Power × 100 **(A, B, C, D, E)** and power × 400 **(A1, B1, C1, D1, E1)**. Panel **(F)** Column charts for the caspase 3-positive stained area. Data from six mice are graphically presented as boxplots, demonstrating the median value and the quartiles. [*] *versus* saline control and [#] *versus* colon cancer control at *p* < 0.05.

Figure 10 shows immunostaining for NFκB. Immunostained colon sections from the saline group showed negative staining (19.2 ± 3.8; Panels 10A and 10A1), whereas sections from the colon cancer control group showed a strong reaction within a hyperplastic colon (56.4 ± 8.1; Panels 10B and 10B1). Immunostained colon sections from the capecitabine group showed a mild–moderate positive reaction (43.8 ± 3.4; Panels 10C and 10C1), whereas the betanin group showed a moderate reaction (54.38 ± 5.5; Panels 10D and 10D1). The photomicrograph of immunostained colon sections from the betanin/capecitabine group showed minimal reaction compared to other groups (36.33; Panels 10E and 10E1). Panel 10F represents the mean area of staining in the groups and shows that the betanin/capecitabine group produced a significant decrease in immunostaining compared (*p* < 0.05) to both the colon cancer group and betanin *per se* group.

## 4 Discussion

Increased consumption of fruits and vegetables is a very effective strategy for enhancing antioxidant intake and decreasing oxidative stress and may result in a reduction in the risk of developing chronic diseases, such as cancer (Song et al., 2010).

In the current study, capecitabine *per se* controlled tumor development pathologic parameters. Cancer chemotherapies are non-invasive agents that lead to delaying, inhibition, or reversing carcinogenesis. Despite that, various chemotherapies have been established, and there are still high numbers of recorded mortalities and morbidities among cancer patients (Kundu et al., 2006). Hence, natural agents with potential antitumor and anti-inflammatory activities are very promising as adjuvant therapies with standard chemotherapies. Hence, in this research article, we focused on exploring the chemotherapeutic potential of the betanin/capecitabine combination versus each *per se* regimen.

Our study revealed histopathological and immunohistochemical changes in the current chemically induced colon cancer mouse model. ACF were originally recognized on the colonic mucosa of the rodents subjected to chemical colorectal carcinogens and have been long considered preneoplastic lesions (Mori et al., 2005). In the present study, DMHZ was employed to induce colon cancer in mice. DMHZ induced various pathologic manifestations such as crowded and proliferating tubular glands, which were characterized by irregular sizes and shapes in addition to distortion in crypts. The mucosal lining demonstrated hyperchromatic nuclei accompanied by severe levels of inflammatory infiltration, as shown in the lamina propria, submucosa, and muscularis layer. Importantly, these pathologic manifestations were partly alleviated in groups receiving the betanin/capecitabine combination or each *per se* therapeutic intervention. These results are supported by those of similar studies using chemical carcinogens in mouse *in vivo* experiments (Abd El-Fadeal et al., 2021; Mohamed et al., 2022).

On the other hand, Feulgen staining is widely used to demonstrate the DNA concentration in tissue specimens. Our results showed a moderate degree of magenta of the Feulgen reaction, reflecting the DNA concentration in the colon specimens of the saline group accompanied by normally distributed columnar epithelia in the colon crypts. Moreover, the significant decrease in magenta in the colon cancer control group reflects DNA damage that was partly restored in groups receiving various treatment strategies. It is known that chemical carcinogens modify the molecular structure of DNA, produce genetic errors, and lead to mutations throughout DNA synthesis. The formation of DNA adducts results in the activation of proto-oncogenes or inactivation of tumor suppressor genes, which is assumed as a tumor initiator step (Hursting et al., 1999).

Quantitative analysis of data on the current colon specimens stained with PAS showed different extents of PAS staining among the study groups, and the greatest difference was recorded between the colon cancer control group and the saline group. Similar results were obtained previously (Mondal et al., 2014; Bahr et al., 2021) as the authors documented a decrease in PAS staining in the hyperplastic colons affected by a chemical carcinogen compared to normal colon samples. These results correspond with the results obtained by other authors (Danquah et al., 2017; Kasprzak et al., 2019). The PAS technique is a very versatile and commonly utilized technique for demonstrating mucin, carbohydrates, and glycoproteins. PAS staining is sensitive particularly for detecting neutral mucins and acid mucins, which incorporate substantial concentrations of sialic acid (Ullah, 2012).

Mucins are complex carbohydrates excreted by the epithelia and connective tissues. Mucin glycoproteins play a crucial role in intestinal protection from injuries; however, the protective mechanism and probable relationship between the structure and function of mucins are only partly known. From a structural point of view, purified intestinal mucins are large-molecular weight glycoproteins of different compositions according to the region and developmental stage. Mucin glycoproteins are implicated in the pathology of epithelial malignancy (Niv, 1994). Modifications in mucin expression patterns have been described in carcinomas and their precursor lesions (Hakomori, 1989).

In the current study, NFκB expression in the colon was elevated in the DMHZ-induced colon cancer control group; this was evident from the mRNA expression and strong immunohistochemical reaction. The mechanisms of NFκB activation in colon cancer are yet to be completely described. Many factors can trigger this activation, such as inflammation mediators, bacterial products, and reactive oxygen species (Baeuerle, 1991; Barnes and Karin, 1997). Indeed, the activation of NFκB in tumors is thought to be associated with malignancy (Raziuddin et al., 1997).

The current results demonstrated the upregulated expression and protein levels of inflammatory mediators (NFκB, TNF-α, IL-1β, and IL-6). Inflammation is mediated through accumulating different immune and inflammatory cells and inflammatory molecules. The interaction between these cells and cytokines results in generating signals that encourage the growth and progression of tumor cells. There is a clearly identified relationship between inflammation and colon cancer, and factors that initiate inflammation can establish colon cancer (Janakiram and Rao, 2014).

Betanin can, at least partly, through anti-inflammatory activity, abate these inflammatory mediators. A recent research paper documented that using gamma-tocopherol and aspirin synergistically defeats colitis-associated colon carcinogenesis and inhibits the growth of human colon cancer cells (Liu et al., 2023). Similarly, it was reported that decreased colon cancer mortality was observed with the regular use of NSAIDs (Zell et al., 2009). In observational studies, aspirin and non-steroidal anti-inflammatory drugs decrease the colon cancer incidence (Nan et al., 2015). Therefore, agents with anti-inflammatory activities are very promising as adjuvant therapies with standard chemotherapies.

The current results demonstrated upregulated expression levels of cyclin D1. Cyclins are chief regulating factors for the progression of the cell cycle and tumor survival. In particular, cyclins D (cyclin D1, cyclin D2, and cyclin D3) are vital intermediaries between proliferation pathways and the cell cycle machinery in the nucleus. Dysregulated expression of cyclins leads to impairing cancer development and carcinogenesis (Pawlonka et al., 2021).

Substantial attention has been focused on the use of beetroot extract or its ingredients for dietary supplementation for preventing carcinogenesis (Gescher et al., 1998; Lee et al., 2004; Stintzing and Carle, 2004; Pan and Ho, 2008; Song et al., 2010). This evidence depends on its apparent capacity to control overoxidative stress that initiates and aggravates cancer and the predominant agreement that long-term exposure to fine amounts of diets rich in antioxidants has cancer chemopreventive potential (Stanner et al., 2004; Azeredo, 2009; Boivin et al., 2009). Betanin has anti-inflammatory activity and suppresses NFκB in rats subjected to acute renal injury (Tan et al., 2015). Similarly, Reddy et al. showed that betanin suppressed 97% of cyclooxygenase-2 (COX-2) enzyme activity (Reddy et al., 2005).

Betanin was confirmed for its anticancer effects and showed significant anticancer effects in human hepatic cell lines (Krajka-Kuźniak et al., 2013), inhibited inflammatory cytokines in microglial cells (Ahmadi et al., 2020), and induced cytotoxicity in U87MG human glioma cells (Salimi et al., 2021). Betanin also inhibits the growth of the breast cancer cell line MCf7 (Reddy et al., 2005) and initiates apoptosis in Caco-2 colon cancer cells (Zielińska-Przyjemska et al., 2016). In addition, betanin inhibits the proliferation of the human colon cancer cell line HCT116 (Reddy et al., 2005). Betanin significantly reduced tumor multiplicity and tumor load after oral administration in female A/J mice (Zhang et al., 2013) but was not tested previously in combination with capecitabine. Hence, the identification of natural compounds that are effective and safe combined with conventional chemotherapeutic drugs can be an effective strategy to combat colon cancer and improve the therapeutic outcome.

## 5 Conclusion

The present study highlighted for the first time that betanin augments the anticancer effect of capecitabine and provides evidence that the mechanism was, at least partly, mediated via its strong anti-inflammatory activity besides the downregulation of cyclin D1 expression.

## Data Availability

The raw data supporting the conclusions of this article will be made available by the authors, without undue reservation.
