# Supplementary material for: Chemotherapeutic potential of betanin/capecitabine combination targeting colon cancer: experimental and bioinformatic studies exploring NFκB and cyclin D1 interplay
Source: Front Pharmacol. 2024 Apr 5;15:1362739. doi: 10.3389/fphar.2024.1362739 (PMC11026609; doi:10.3389/fphar.2024.1362739)
Supplement: Supplementary file 1 [file DataSheet1.PDF]

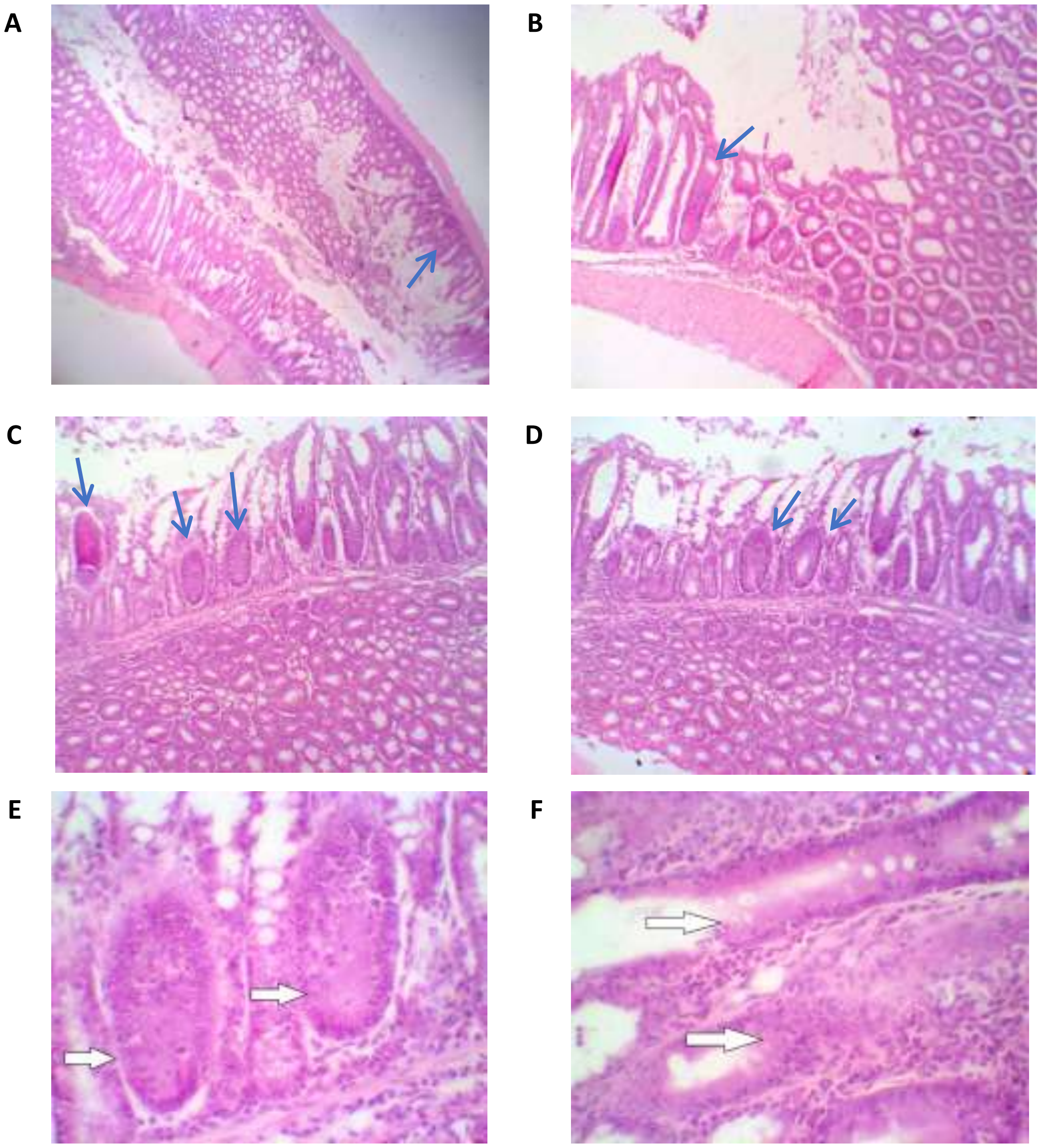

Supplementary figure 1: Hematoxylin and eosin staining for colon sections showing aberrant crypts. Image A, B, C & D: images show aberrant crypts (blue arrows). Image E & F: show high power images for aberrant crypts (thick white arrows) in the mice received dimethylhydrazine.
